# A *Litopenaeus vannamei* Hemocyanin-Derived Antimicrobial Peptide (Peptide B11) Attenuates Cancer Cells’ Proliferation

**DOI:** 10.3390/molecules23123202

**Published:** 2018-12-05

**Authors:** Shangjie Liu, Jude Juventus Aweya, Liyuan Zheng, Fan Wang, Zhou Zheng, Mingqi Zhong, Jingsheng Lun, Yueling Zhang

**Affiliations:** 1 Department of Biology and Guangdong Provincial Key Laboratory of Marine Biotechnology, Shantou University, Shantou 515063, China; 15sjliu1@stu.edu.cn (S.L.); jjaweya@stu.edu.cn (J.J.A.); zhengly@wibe.ac.cn (L.Z.); wangfan@stu.edu.cn (F.W.); zhengzhou@stu.edu.cn (Z.Z.); mqzhong@stu.edu.cn (M.Z.); jslun@stu.edu.cn (J.L.); 2 STU-UMT Joint Shellfish Research Laboratory, Shantou University, Shantou 515063, China

**Keywords:** *Litopenaeus vannamei*, hemocyanin-derived peptide, B11, cell proliferation, apoptosis, mitochondria

## Abstract

Antimicrobial peptides play important roles in the immune response to pathogens and tumor cells; for this reason, they are being exploited for therapeutic use. In this study, we describe a *Litopenaeus vannamei* hemocyanin-derived peptide, denoted B11, which shares similar features with other anticancer peptides and attenuates the proliferation of cancer cells. Cell viability assay revealed that B11 significantly inhibited the proliferation of human cervical (HeLa), human hepatocellular carcinoma (HepG2), and human esophageal cancer (EC109) cancer cell lines, but not normal liver cell lines (T-antigen-immortalized human liver epithelial (THLE) cells or THLE-3), by inducing morphological changes, nuclear condensation, and margination, features which are indicative of apoptosis. Besides, peptide B11-induced apoptosis was confirmed by isothiocyanate-labeled Annexin V/propidium iodide (Annexin V-FITC/PI) double staining of HeLa cells. Moreover, cell uptake studies, confocal microscopy, and Western blot analysis revealed that rhodamine-labeled B11 permeated HeLa cells and localized to the mitochondria, causing mitochondria dysfunction through lost mitochondrial membrane potential, which consequently triggered the induction of apoptosis. Increased expression levels of caspase-9, caspase-3, and Bax (Bcl-2-associated X) proteins, coupled with a decrease in Bcl-2 (B-cell lymphoma 2) protein, confirmed that peptide B11 induced apoptosis via the mitochondrial pathway. Thus, the hemocyanin-derived peptide, B11, inhibits the proliferation of cancer cells by causing mitochondrial dysfunction and inducing apoptotic cell death, for which reason it could be explored as an anticancer peptide.

## 1. Introduction

Hemocyanin (HMC) is a respiratory protein, which is also reported to be multi-functional, and thus plays essential roles in mollusk and arthropods [1,2]. A growing number of immune-related functions have been ascribed to hemocyanin [3,4], including phenoloxidase [5], antiviral [6], agglutinative [7,8], anticancer [9], reaction with anti-human Ig (immunoglobulin) as an antigen [10], hemolysin activity [11], and as an immune-enhancement protein in shrimp [12]. Moreover, in different crustaceans, hemocyanin is reported to generate antimicrobial peptides (AMPs) in response to microbial challenge [13]. Destoumieux-Garzón et al. reported that the C-terminal fragment of hemocyanin from penaeid shrimps *Penaeus vannamei* and *Penaeus stylirostris* had broad antifungal activities [14]. An antibacterial peptide with 16 amino acid residues was also found in the plasma of freshwater crayfish *Pacifastacus leniusculus* [15]. Similarly, we previously found an 18.4-kDa fragment of hemocyanin with antimicrobial activity in *Litopeneus vannamei* infected with *Vibrio parahaemolyticus* [16]. Generally, AMPs are small cationic peptides characterized by positive charges and hydrophobic amino acids, as well as amphipathic features [17]. Since AMPs are positively charged, they are able to bind to negatively charged bacteria cell membranes, resulting in the disruption of the membrane and bacteria death [18]. These features and properties of AMPs makes them important components of the innate immune system in a variety of organisms, including plants and animals [19,20].

Several recent studies have shown that AMPs also have anticancer activity [21]. For instance, Rodrigues et al. reported that a cream that is mixed with the AMP gomesin, and used as a topical drug to smear over the external surface of tumors, successfully treated intradermal and intraepithelial cancers [22]. A synthetic 21-mer AMP (Epinecidin-1) from grouper (*Epinephelus coioides*) has been reported to have in vitro antitumor activity against human fibrosarcoma cells (HT1080) [23]. Similarly, the pleurocidin family of AMPs (NRC-03 and NRC-07) derived from *Atlantic flounder* were found to kill breast carcinoma cells, including drug-resistant and slow-growing breast cancer cells [24]. Interestingly, our recent studies involving the screening of *L. vannamei* hemocyanin identified 20 potential AMPs ranging from 1.5 to 1.9 kDa [25]. While the antibacterial activities of these hemocyanin-derived peptides have been ascertained, whether or not these peptides also have anticancer effects is not known.

In the current study, we report on the antiproliferative and potential anticancer activity of one of these *L. vannamei* hemocyanin-derived AMPs (designated B11). Peptide B11 could inhibit the proliferation of three cancer cell lines by permeabilizing, entering, and inducing apoptotic cell death. Given the properties exhibited by peptide B1, it could be used for anticancer agents, while the knowledge gained from this study could provide the basis for developing therapeutic peptides from marine resources into anticancer therapeutic agents.

## 2. Results

### 2.1. Synthesis and Characterization of Peptides

**Figure 1:**
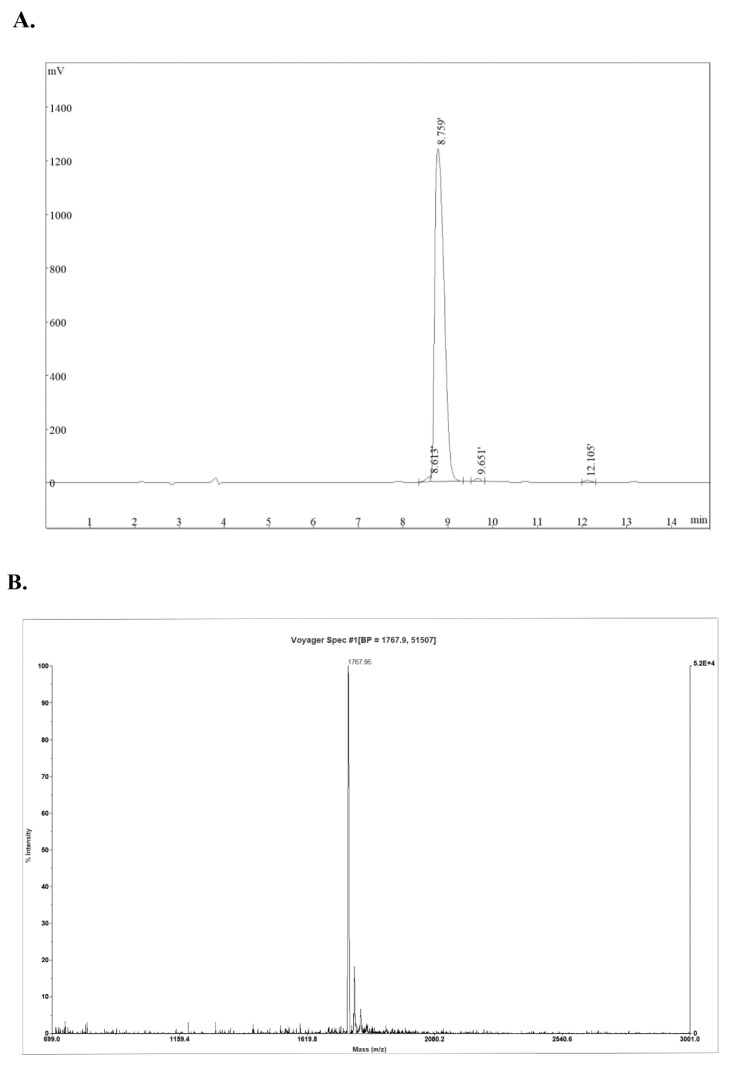
Reverse phase high performance liquid chromatography (RP-HPLC) and matrix-assisted laser desorption/ionization time-of-flight mass spectrometry (MALDI-TOF-MS) analysis of *L. vannamei* hemocyanin-derived antimicrobial peptide B11. (**A**) Chromatographic profile of purified peptide B11 products, (**B**) MALDI-TOF-MS spectra of purified peptide B11.

The *L. vannamei* hemocyanin-derived antimicrobial peptide (B11) was synthesized manually via solid phase peptide synthesis (SPPS) using the Fluorenylmethyloxycarbonyl/*tert*-butyloxycarbonyl (Fmoc/tBu) strategy [26]. The purity and molecular mass of the successfully purified peptide B11 using the SPPS method was ascertained by reverse phase high performance liquid chromatography (RP-HPLC) and matrix-assisted laser desorption/ionization time-of-flight mass spectrometry (MALDI-TOF-MS) analysis (Figure 1). 

### 2.2. Effect of Peptide B11 on Cancer Cells’ Proliferation

**Figure 2:**
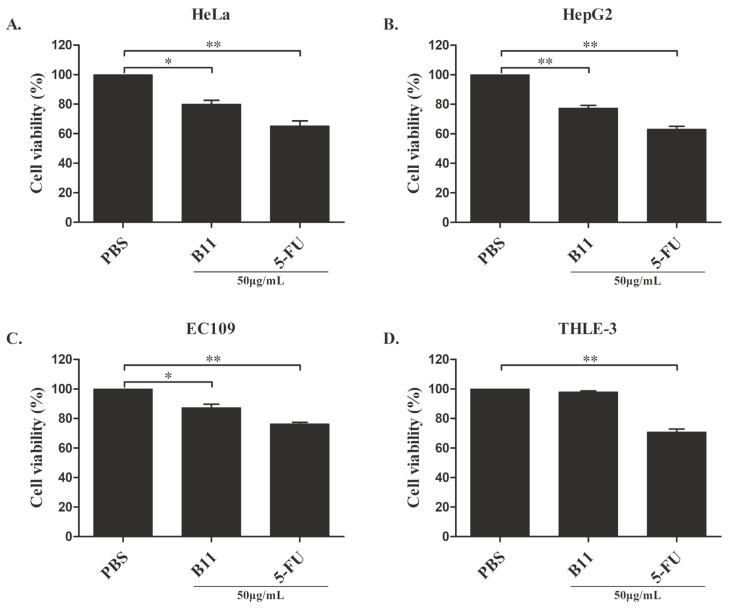
Inhibitory effects of peptide B11 on growth of immortalized cancer and non-cancer cells. The cancer cell lines (**A**) human cervical cancer (HeLa) cells, (**B**) human hepatocellular carcinoma (HepG2) cells, (**C**) human esophageal cancer (EC109) cells, and immortalized normal human liver cells (**D**) THLE-3 cells (T-antigen-immortalized human liver epithelial (THLE) cells) were grown for 24 h in the presence of 50 μg/mL of peptide B11. Cell proliferation was analyzed using the MTS assay with PBS used as the negative control, while 5-fluorouracil (5-FU) was used as the positive control. Data represent means ± SD for three independent experiments (*n* = 3). Significant difference relative to control (PBS-treated cells) were determined by one-way ANOVA and indicated by asterisks (* *p* < 0.05, ** *p* < 0.01).

The antiproliferative activity of peptide B11 against some cancer cell lines, including HeLa cells (human cervical cancer cells), HepG2 cells (human hepatocellular carcinoma cells), and EC109 cells (human esophageal cancer cells) was examined. When the cell proliferation or viability following treatment with peptide B11, 5-fluorouracil (5-FU), or PBS (Phosphate-buffered saline) was determined using the MTS assay (Figure 2), it was observed that the proliferation of all three cancer cell types was significantly decreased 24 h post-treatment with peptide B11 or with the anticancer drug 5-FU compared with PBS. For instance, peptide B11 significantly (*p* < 0.05) decreased HeLa cells viability by 20.0% (Figure 2A), while that of HepG2 cells decreased by 23.0% (Figure 2B) and EC109 cells decreased by 13.0% (Figure 2C) relative to PBS treatment. On the contrary, peptide B11 had no significant effect on the proliferation of normal liver cell lines (THLE-3) (Figure 2D), as treatment with B11 or PBS for 24 h had almost the same cell viability (97.92% and 100%, respectively). Thus, these results suggest that peptide B11 selectively inhibits the in vitro proliferation of only cancer cell lines, and could potentially be used as an antitumor agent.

### 2.3. Peptide B11 Induces Apoptosis in HeLa Cells

**Figure 3:**
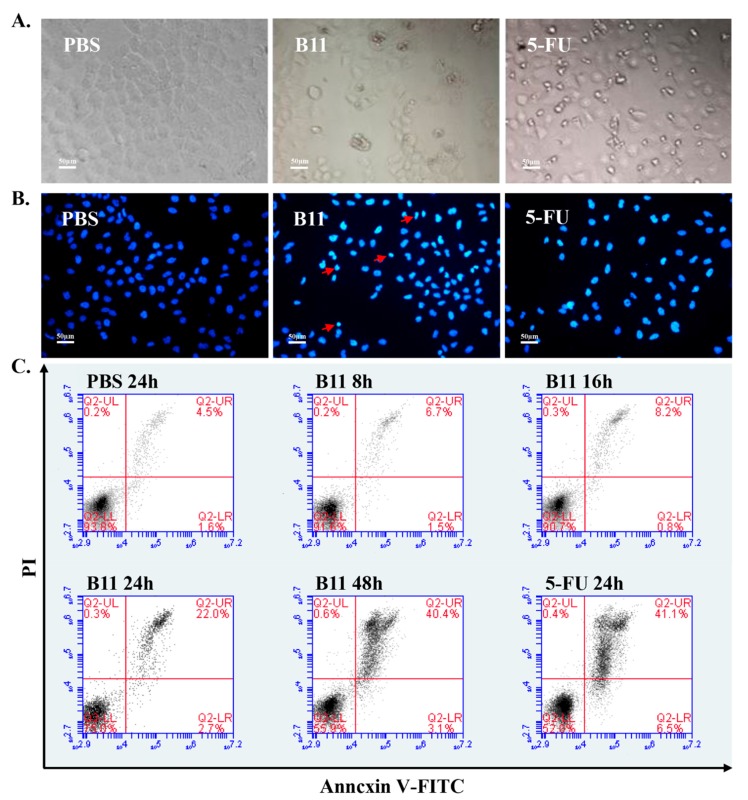
Peptide B11 affects the cell morphology and induces the apoptosis of HeLa cells. (**A**) Changes in cell morphology following treatment with PBS, peptide B11, and 5-FU for 24 h. Micrographs were obtained using an inverted microscope (20×). (**B**) Changes in cell nuclei morphology following treatment with PBS, peptide B11, and 5-FC for 24 h. The 4,6-diamidino-2-phenylindole dihydrochloride (DAPI) stained nuclei were observed with a fluorescence microscope (20×). (**C**) Flow cytometric analysis of apoptosis in HeLa cells after 8 h to 48 h of treatment with PBS, peptide B11, and 5-FU, and staining with Annexin V/propidium iodide (Annexin V/PI). Quadrants: lower-left represent live cells (Annexin V negative/PI negative); lower-right represent early apoptotic/primary apoptotic cells (Annexin V positive/PI negative); upper-right represent late apoptotic/secondary apoptotic cells (Annexin V positive/PI positive); upper-left represent necrotic cells (Annexin V negative/PI positive). The numbers in the respective quadrants indicate the percentage of cells present in that area. Data shown represent one of three independent experiments.

The induction of apoptosis induction and changes to cell morphology were examined to ascertain if this could be responsible for the antiproliferative effect of peptide B11. As shown in Figure 3A, HeLa cells treated with peptide B11 (50 μg/mL) for 24 h were changed morphologically, including cell shrinkage and rounding up. A similar observation was found in 5-FU-treated cells, but not in the PBS-treated cells. In addition, the typical characteristics of apoptosis, including nuclei shrinkage, condensation, margination and the formation of apoptotic body-like vesicles were observed in cells 24 h post-treatment with peptide B11 and 5-FU, but not in PBS (Figure 3B). Moreover, flow cytometry analysis with Annexin V/propidium iodide (Annexin V/PI) staining revealed that the ratio of viable cells to total cells gradually decreased in peptide B11-treated cells, while there was a time-dependent gradual increase in the percentage of late apoptotic cells (Figure 3C). In fact, the average percentage of apoptotic cells in peptide B11-treated cells was 40.4% at 48 h post-treatment, which is similar to that observed in the 5-FU treated cells (41.1%) at the same time point. These results suggest that peptide B11 exerts a proapoptotic effect on cancer cells, which is synonymous with 5-FU, and that this might account for the antiproliferative effects of peptide B11 on HeLa cells.

### 2.4. Physicochemical Properties of B11 and Cell Permeabilization Analysis

**Figure 4:**
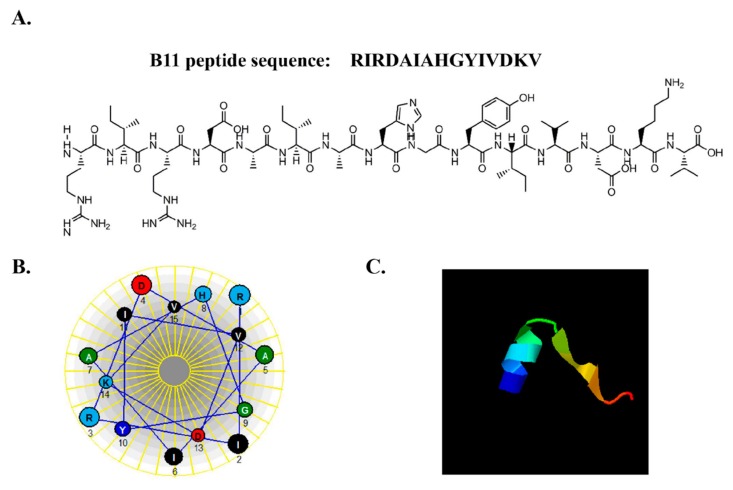

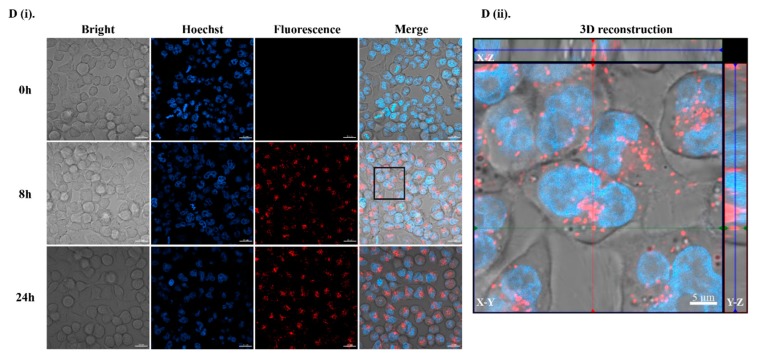
Predicted structure of peptide B11 and cell uptake analysis. (**A**) Amino acid sequence and primary structure of peptide B11. (**B**) The Schiffer–Edmundson helical wheel representation of peptide B11, showing the helical wheel projects and arrangement of amino acid residues counting from the amino (*N*) terminus. (**C**) Predicted 3D modeled structure of peptide B11. (**D**) Uptake of rhodamine-labeled B11 by HeLa cells. Cells were treated with rhodamine-labeled B11 at the indicated time points (0 h, 8 h, and 24 h), stained with Hoechst 33342, and then observed under a confocal microscope (scale bar = 20 µm).

Peptide B11 is located within the copper-containing domain of the large molecular weight *L. vannamei* hemocyanin protein (GeneBank accession No.: CAB85965.1), and is composed of 15 amino acids, half of which are hydrophobic (Figure 4A). The physicochemical properties of peptide B11, including molecular weight, total hydrophobic ratio, hydrophobicity, hydrophobic moment, average hydropathy value, molar extinction coefficient, charge and isoelectric point are summarized in Table 1. Peptide B11 has a molecular weight of 1767 Da, with a net positive charge, and a total hydrophobic ratio of 46%. Modeling using the Schiffer–Edmundson helical wheel on the DNAstar (Lasergene 7.1, Madison, WI, USA) program revealed that peptide B11 has an amphipathic α-helical conformation (Figure 4B), while three-dimensional (3D) model prediction using the I-TASSER program (http://zhanglab.ccmb.med.umich.edu/I-TASSER) showed that it has α-helical and one β-sheet structure (Figure 4C). Thus, given the size of peptide B11, its net positive charge, α-helix, and β-sheet structure, as well as other features that are typical of therapeutic peptides and/or AMPs [27,28], it should be able to permeable cells [29].

Cell uptake studies were carried out to determine whether peptide B11 could indeed penetrate cell membranes. Confocal microscopy analysis showed that rhodamine-labeled B11 was readily taken up by HeLa cells, with the number of fluorescent cells higher at 24 h post-treatment compared to 8 h, suggesting that peptide B11 penetrates cells time-dependently (Figure 4D(i)). Moreover, analysis of the X–Z and Y–Z sections of the three-dimensional (3D) reconstructed confocal images confirmed that peptide B11 was found inside the cytoplasm of cells (Figure 4D(ii)), further suggesting that as a cationic peptide, B11 could permeate cells to exert its effects.

### 2.5. Peptide B11 Induces Mitochondrial-Dependent Apoptotic Cell Death in HeLa Cells through Lost of Mitochondrial Membrane Potential

**Figure 5:**
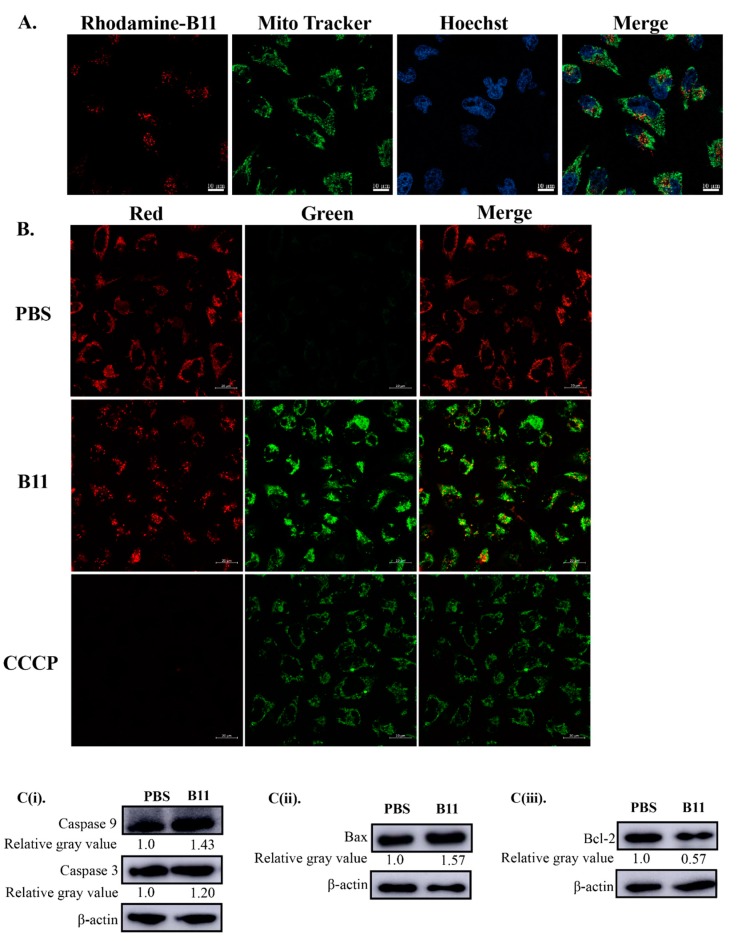
Localization of peptide B11 in the mitochondria, its effect on mitochondrial membrane potential (∆Ψm), and apoptosis induction in HeLa cells. (**A**) Microscopic images showing the intracellular localization of rhodamine-labeled B11 in HeLa cells. Cells were treated with 50 μg/mL of rhodamine-labeled B11 for 8 h, washed with PBS and stained with 200 nM of MitoTracker Green. Images were captured with a confocal microscope under a 40× objective (scale bar = 10 µm). (**B**) Mitochondrial membrane potential (∆Ψm) of HeLa cells treated with peptide B11. Cells were treated for 24 h with peptide B11 (50 μg/mL) and PBS (0.01 M, pH 7.4), followed by staining with 5,5′,6,6′-tetrachloro-1,1′,3,3′-tetraethylbenzimidazole-carbocyanide iodine (JC-1) working solution and incubated for 20 min at 37 °C protected from light. Images were observed under confocal microscopy (scale bar = 20 µm). For positive control, cells were treated with 10 µM of carbonyl cyanide m-chlorophenylhydrazone (CCCP). Red fluorescence represents the mitochondrial aggregate form of JC-1, indicating an intact mitochondrial membrane potential. Green fluorescence represents the monomeric form of JC-1, indicating dissipation of the ∆Ψm. (**C**) Immunoblots of (**i**) caspase-9 and caspase-3, (**ii**) Bax, and (**iii**) Bcl-2 protein levels in peptide B11-treated HeLa cells analyzed by Western blot. Cell lysates from HeLa cells treated with peptide B11 (50 μg/mL) or PBS (0.01 M, pH 7.4) for 24 h were analyzed using the appropriate antibodies, with β-actin used as a loading control. Numbers below the blots represent the relative gray values determined using ImageJ program.

The integrity of mitochondrial function is crucial for the maintenance of cell viability [30]. Thus, given that peptide B11 has a net positive charge, induces apoptosis, and could permeate cells, we went about determining the effect of peptide B11 on the mitochondria. First, confocal microscopy revealed that rhodamine-labeled B11 colocalized with MitoTracker in the mitochondria of HeLa cells (Figure 5A). Moreover, peptide B11 not only localized to the mitochondria, but was able to cause mitochondrial dysfunction in the form of a loss in mitochondrial membrane potential (Figure 5B). In addition, Western blot analysis was used to further examine the expression of proteins involved in the mitochondrial-dependent apoptosis pathways following treatment with peptide B11. As shown in Figure 5C(i), at 24-h post-treatment of HeLa cells with B11 (50 μg/mL), there was an increase in the protein expression levels of caspase-3 (an executioner caspase in the apoptosis cascade) and caspase-9 (an initiator caspase in the mitochondrial-dependent or intrinsic apoptosis pathway) compared to PBS treatment. Besides, there was an increase in the protein expression level of proapototic Bax (Figure 5C(ii)) and a decrease in the protein expression level of prosurvival Bcl-2 (Figure 5C(iii)), two Bcl-2 family members that play a central role in regulating changes in mitochondrial outer membrane permeability [31,32]. All of these results indicate that peptide B11 was able to cause mitochondrial dysfunction and induced apoptosis via the mitochondrial-dependent pathway.

## 3. Discussion

Several recent studies have reported on the development and sophisticated resistance being mounted by cancer cells and bacteria against most of the traditional drugs [33,34,35]. Therefore, researchers are exploring alternative more efficacious drugs, with more attention being drawn toward the use of naturally occurring antimicrobial peptides (AMPs). Several AMPs have so far been evaluated in both preclinical and clinical studies [36,37], with data suggesting that AMPs could substitute traditional antibiotics in combatting microbial infections, especially multidrug-resistant microbes [17,38]. Most importantly, some AMPs have been shown to have antitumor activity [39,40]. In the current study, an in silico predicted AMP (designated peptide B11) derived from the hemocyanin protein of the shrimp *L. vannamei*, was synthesized manually via solid phase peptide synthesis, and shown to significantly inhibit the proliferation of cancer cells. The antiproliferative effect of peptide B11 is because it causes mitochondrial dysfunction and induces apoptosis, therefore suggesting its anticancer potential. The research approach that was used in this study implies that bioinformatics prediction tools could be leveraged to explore anticancer therapeutic agents followed by cell-based validation. 

While a number of studies have explored the discovery of AMPs, and studied their structures and mechanisms of action [41,42], recent reviews have reported on the anticancer activities and efficacy of AMPs from terrestrial animals and plants [21,43,44,45]. The shrimp *L. vannamei* is reported to generate different AMPs from hemocyanin, both naturally as part of the innate immune system and in response to specific pathogen challenge [13,14,46]. The antimicrobial activities of these shrimp hemocyanin-derived AMPs have been demonstrated [25]; however, their effect on cell proliferation and potential use as antiproliferative or antitumor agents has not been explored. 

In this study, the antitumor potential of a 15 amino acids hydrophobic cationic antimicrobial peptide, peptide B11, which is derived from the large molecular weight hemocyanin protein of *L. vannamei*, and has α-helical and β-sheet structure, was explored. The structure and physicochemical properties of AMPs are reported to play a significant role in their ability to induce apoptosis in cancer cells [21,43]. Cationic AMPs are able to physically associate with the negatively charged membranes of cancer cells, destabilizing the lipid membrane and subsequently binding to intracellular targets, resulting in cell death [24]. On the other hand, AMPs rich in hydrophobic amino acids are able to insert into cell membranes to form a stable structure, thereby disrupting the cell membrane and forming pores, leading to changes in cell membrane charge and therefore interfering with cell death pathways [21]. Given that the features of peptide B11 (Table 1 and Figure 4A) are synonymous with cationic anticancer peptides, this suggests that peptide B11 might share similar properties and/or functions. Since the outer membranes of cancer cells are negatively-charged due to an abundance of anionic molecules, while the membranes of noncancerous or normal cells are neutral [44,47], cationic peptides such as B11 could exert antiproliferative effects targeted at only cancer cells, due to this membrane charge difference. In the current study, rhodamine-labeled B11 was able to permeate HeLa cells (Figure 4D), just as other anticancer peptides [40,48], and localized to the mitochondria (Figure 5A), where it induced a loss of mitochondrial membrane potential (Figure 5B), and consequently mitochondrial dysfunction. Dysfunctional mitochondria might eventually lead to the induction of apoptosis via the mitochondrial-dependent pathway [49].

In the apoptosis pathway, caspases (cysteine proteases) act in concert in a cascade to trigger apoptosis [50,51]. The apoptosis-related caspases are generally divided into initiator caspases (including caspase-2, caspase-8, caspase-9, and caspase-10), and effector or executioner caspases (including caspase-3, caspase-6, and caspase-7) [51,52,53]. As the initiator caspase in the mitochondrial-dependent apoptotic pathway [54], activated caspase-9 in turn activates caspase-3/7 potentiating the apoptosis cascade, and thereby catalyzing the cleavage of many key cellular proteins that commit cells to apoptosis. To ascertain that there was an induction of apoptosis due to the mitochondrial dysfunction, an increase in the protein expression levels of caspase-9 and caspase-3 (determined by Western blot) was observed following the treatment of HeLa cells with peptide B11 (Figure 5C(i)). Since the Bcl-2 family proteins, which govern mitochondrial outer membrane permeability, are either proapoptotic (Bax, BAD, Bak, Bok, etc.) or antiapoptotic (Bcl-2, Bcl-xL, Bcl-w, etc.) [55], we went on to determine changes in the protein expression of Bcl-2 and Bax, given their important role in mitochondrial-dependent apoptosis [31,56]. Interestingly, peptide B11 treatment caused a decrease in antiapoptotic Bcl-2 protein, but an increase in proapototic Bax in HeLa cells (Figure 5C(ii),(iii)). This observation is similar to that previously reported by Wang et al., where an oligopeptide from *Sepia* ink induced the apoptosis of lung cancer cells via the mitochondrial pathway [57]. The antiapoptotic Bcl-2 family proteins prevent cell death induced by various apoptotic stimuli by inhibiting the release of mitochondrial cytochrome C, and inhibiting the activation of caspase-9 and caspase-3 [58]. Thus, a decrease in the expression of Bcl-2 protein coupled with an increase in the expression of Bax often facilitates apoptosis [59,60]. The studies presented thus far provide evidence that suggest peptide B11 exerts an antiproliferative effect in cancer cells by targeting the mitochondria, causing mitochondrial dysfunction through a loss in membrane potential, and consequently inducing mitochondrial-dependent apoptosis. 

## 4. Materials and Methods 

### 4.1. Peptide Synthesis

Peptide B11 (RIRDAIAHGYIVDKV) and rhodamine-labeled B11 were synthesized by a commercial company (Scilight Biotechnology, Beijing, China) using the solid-phase procedure as reported by Huertas et al. [61]. Briefly, 9-fluorenylmethoxycarbonyl amino acid (Fmoc amino acid) and 2,6-dichlorobenzenoylchloride (DCB) were added to the resin to attach the first amino acid. The deprotection was conducted by adding piperidine to remove the protection group, and then the activated amino acid was attached to the peptidyl resin with agitation to couple the next residue. The cycle of deprotection and coupling was repeated until the target peptide was completely synthesized. Rhodamine-labeling was carried out after the last deprotection and coupling step. The peptide was then cleaved from the resin with trifluoroacetic acid (TFA). The disulfide bridge in the peptide was formed by DMF, and C-terminal amidation was conducted by the amidating enzyme. The synthetic peptides were purified by reversed-phase high performance liquid chromatography (RP-HPLC, Omaha, NE, USA) with an Agela C18 column (Agilent, CA, USA). The purity and molecular masses of the purified synthetic peptides were determined using RP-HPLC and MALDI-TOF mass spectrometry (Bruker Daltonics, Bremen, Germany).

### 4.2. Cell Culture

Human cervical cancer cells (HeLa cells), human hepatocellular carcinoma cells (HepG2 cells), human esophageal cancer cell (EC109 cells), and normal liver cell lines (THLE-3 cells) were kind gifts from Dr. En Min Li of Shantou University Medical College, Shantou University, China. All of the cell lines were cultured in Dulbecco’s Modified Eagle Medium (DMEM, Thermo Fisher Scientific Inc., Waltham, MA, USA) supplemented with 10% fetal bovine serum (FBS, Gibco, Carlsbad, CA, USA) and 1% penicillin/streptomycin (Gibco), and then maintained in a 5% CO_2_ incubator at 37 °C.

### 4.3. Cell Proliferation Assay

Cell proliferation was determined using the MTS assay as reported by Kong et al. [62]. Briefly, 5 × 10^3^ cells were seeded onto 96-well plates overnight. Next, cells were treated with 50 μg/mL of peptide B11 or 50 μg/mL 5-fluorouracil (5-FU) as a positive control, with PBS (0.01 M, pH 7.4) used as the negative control. After 24 h, 20 µL/well of CellTiter 96 AQ_ueous_ One Solution (MTS) solution (Promega, Madison, WI, USA) was added and incubated at 37 °C for 3 h. Optical density (OD) was measured using a microplate reader (BioTek, Winooski, VT, USA) at 490 nm. The viability rate was calculated as, cell viability% = OD_B11_/OD_PBS_ × 100%. Triplicate samples were analyzed, and data were represented as means ± standard error (SD). Experiments were repeated at least three times and the *p*-values were determined using Student’s *t*-test.

### 4.4. Cytological Effect of Peptide B11 on HeLa Cells

To examine the cytological effects of peptide B11 on cells, HeLa cells were plated onto 96-well plates (5 × 10^3^/well) overnight, followed by treatment with peptide B11 (50 μg/mL), 5-FU (50 μg/mL), or PBS (0.01 M, pH 7.4) for 24 h. Next, media was removed, and cells were washed two times with PBS (0.01 M, pH 7.4). Following this, cells were fixed for 20 min with 2.5% glutaraldehyde, and then washed twice with PBS (0.01 M, pH 7.4). The fixed cells were stained for 8 min with 1 μg/mL of 4,6-diamidino-2-phenylindole dihydrochloride (DAPI) at 37 °C. Cells were washed four times with PBS (0.01 M, pH 7.4), and finally examined under a fluorescence microscope (Olympus, Tokyo, Japan) as previously described [63].

### 4.5. Annexin V-FITC/PI Apoptosis Detection Assay

The ability of peptide B11 to induce cell death in terms of apoptosis was determined using flow cytometry with Annexin V-FITC apoptosis detection kit (Beijing Beyotime Corp, Beijing, China) following the manufacturers’ instructions. Briefly, HeLa cells were plated onto 24-well plates (1 × 10^5^/well) overnight. Next, cells were treated with peptide B11 (50 μg/mL), PBS (0.01 M, pH 7.4), and 5-FU (100 μg/mL) for eight to 48 h. At each time point, cells were washed with PBS (0.01 M, pH 7.4) and re-suspended in 195 μL of binding buffer (10 mM of Hepes/NaOH, pH 7.4, 140 mM of NaCl, 2.5 mM of CaCl_2_); then, five μL of Annexin V-FITC and 10 μL of propidium iodide (PI) were added to stain for 15 min at room temperature in the dark. Cells were analyzed on an Accuri C6 flow cytometer (BD bioscience, SanDiego, CA, USA). 

### 4.6. Prediction of the Structural Characteristics and Features of Peptide B11 

The physicochemical properties of peptide B11 were analyzed using the antimicrobial peptide database web server (APD3, Omaha, NE, USA), while the helical properties of peptide B11 were determined by Schiffer Edmundson wheel modeling using the DNAstar Lasergene 7.1 programme. The 3D structure of peptide B11 was predicted using the I-TASSER program (Version 5.1, zhanglab, Ann Arbor, MI, USA) (http://zhanglab.ccmb.med.umich. edu/I-TASSER).

### 4.7. Cell Uptake Studies

The uptake of peptide B11 by cells was performed according to the method described by Wang et al. [48]. Briefly, HeLa cells (1 × 10^5^) were plated onto a 35-mm glass-bottom dish (In Vitro Scientific, Sunnyvale, CA, USA) overnight, followed by treatment with 50 μg/mL of rhodamine-labeled B11 for eight to 24 h, and placed in a CO_2_ incubator at 37 °C. The media was removed, and the cells were washed three times with PBS (0.01 M, pH 7.4). Following this, cells were stained with one μg/mL of Hoechst 33342 (Beijing Beyotime Corp) for 10 min at 37 °C, before being washed three times with PBS (0.01 M, pH 7.4). Cells were imaged using a confocal microscope (LSM 800, Carl Zeiss, Jena, Germany).

### 4.8. Analysis of Subcellular Localization

HeLa cells (1 × 10^5^) were plated onto a 35-mm glass-bottom dish (In Vitro Scientific) overnight, and then treated with 50 μg/mL of rhodamine-labeled B11 for eight h, and placed in a CO_2_ incubator at 37 °C. The media was removed, and the cells were washed three times with PBS (0.01 M, pH 7.4). Following this, cells were incubated for 30 min with 200 nM of MitoTracker Green (Beijing Beyotime Corp), and then for 10 min with one μg/mL Hoechst 33342 at 37 °C to visualize the mitochondria and nuclei, respectively. Next, the reagents were removed, and the monolayer of cells washed three times with ice-cold PBS before being examined by a confocal laser-scanning microscope (LSM 800, Carl Zeiss, Germany).

### 4.9. JC-1 Dye Staining for Mitochondrial Membrane Potential Analysis 

The loss of mitochondrial membrane potential (∆Ψm) was examined by confocal laser-scanning microscopy (LSM 800, Carl Zeiss, Germany) using 5,5′,6,6′-tetrachloro-1,1′, 3,3′-tetraethylbenzimidazole-carbocyanide iodine (JC-1; Beijing Beyotime Corp) staining. Briefly, HeLa cells were seeded at a density of 1 × 10^5^/well onto a 35-mm glass-bottom dish (In Vitro Scientific). After overnight incubation, cells were treated for 24 h with peptide B11 (50 μg/mL) and PBS (0.01 M, pH 7.4); then, they were placed in a carbon dioxide (CO_2_) incubator at 37 °C. The spent media was removed, and cells washed once with PBS. Next, one mL of complete culture media and one mL of JC-1 working solution were added to each well, and then they were incubated for 20 min at 37 °C protected from light according to the manufacturer’s instructions. After being washed twice with buffer solution, cells were examined using a laser-scanning confocal microscope (LSM 800, Carl Zeiss, Germany) at room temperature. Cells were treated with 10 μM of carbonyl cyanide m-chlorophenylhydrazone (CCCP), which is a protonophore that can cause the dissipation of ∆Ψm, and was used as positive control.

### 4.10. Western Blot Analysis

Western blot analysis was performed as described previously [64]. Briefly, HeLa cells were plated onto six-well plates (1 × 10^6^/well) overnight, and then treated with peptide B11 (50 μg/mL) and PBS (0.01 M, pH 7.4) for 24 h and collected, washed, and pelleted by centrifugation. Cell pellets were lysed with Radioimmunoprecipitation assay (RIPA) buffer (150 mM of sodium chloride, 1% Triton X-100, 1% sodium deoxycholate, 0.1% SDS, 50 mM of Tris–HCl, pH 7.5, and 2 mM of ethylenediaminetetraacetic acid (EDTA)) with protease inhibitor (Roche, Indianapolis, IN, USA). Total protein extracts were separated by sodium dodecyl sulfate polyacrylamide gel electrophoresis (SDS-PAGE) using a 12% SDS-PAGE gel, and then transferred onto a polyvinylidene difluoride (PVDF) membrane with a wet transfer apparatus according to the manufacturer’s instructions (Bio-Rad, Richmond, CA, USA). The membrane was blocked for 1 h with 5% skimmed milk in Tris Buffered Saline with Tween (TBST) (0.1% Tween-20, 20 mM Tris, 0.15 M NaCl, pH 7.4) at room temperature. Next membranes were probed with primary antibodies: anti-caspase-9 antibody (Rabbit monoclonal, Abcam; 1:1000), anti-caspase-3 antibody (Rabbit monoclonal, Abcam; 1:1000), anti-Bax (Rabbit monoclonal, Abcam; 1:1000), anti-β-actin antibody (Rabbit polyclonal, Abbkine; 1:1000) or anti-Bcl-2 antibody (Mouse monoclonal, Abbkine; 1:1000). After being washed three times with TBST, membranes were then incubated with horseradish peroxidase (HRP) linked goat anti-mouse or goat anti-rabbit secondary antibodies (Sigma-Aldrich, St Louis, MO, USA 1:5000) for 1 h at room temperature. Signals were detected by chemiluminescence using an enhanced chemiluminescence (ECL)-detecting reagent, and images were captured using the GE Amersham Imager 600 imaging system (GE, Boston, MA, USA).

## 5. Conclusions

Collectively, the data generated in the current study indicates that antimicrobial peptide B11, which is derived from the large molecular weight hemocyanin protein of *L. vannamei*, has antiproliferative effects on cancer cells, as it was able to cause mitochondrial dysfunction and induce apoptosis. In view of these interesting findings, future studies would explore the antitumor potential of peptide B11 using an animal model.

## 6. Patents

Zhang YL, Liu SJ, Zheng LY and Wang F have filed a patent that relates to the use of the peptide (B11) for tumor suppression.

## Figures and Tables

**Table 1:** Predicted physicochemical properties of peptide B11.

Property	Parameter
Sequence	RIRDAIAHGYIVDKV
Molecular weight (Da)	1767
Total hydrophobic ratio	46%
Charge at pH 7.0	+1
Hydrophobicity <H>	0.333
Hydrophobic moment <µH>	0.119
Average hydropathy value	0.0466
Molar extinction coefficient	1490
Isoelectric point	8.862

## References

[1] Jaenicke E., Föll R., Decker H. (1999). Spider hemocyanin binds ecdysone and 20-OH-ecdysone. J. Biol. Chem..

[2] Paul R.J., Pirow R. (1997). The physiological significance of respiratory proteins in invertebrates. Zoology.

[3] Coates C.J., Decker H. (2016). Immunological properties of oxygen-transport proteins: Hemoglobin, hemocyanin and hemerythrin. Cell Mol. Life Sci..

[4] Coates C.J., Nairn J. (2014). Diverse immune functions of hemocyanins. Dev. Comp. Immunol..

[5] Decker H., Rimke T. (1998). Tarantula hemocyanin shows phenoloxidase activity. J. Biol. Chem..

[6] Nesterova N.V., Zagorodnya S.D., Moshtanska V., Dolashka P., Baranova G.V., Golovan A.V., Kurova A.O. (2011). Antiviral activity of hemocyanin isolated from marine snail *Rapana venosa*. Antivir. Res..

[7] Zhang Y., Wang S., Xu A., Chen J., Lin B., Peng X. (2006). Affinity proteomic Approach for identification of an IgA-like protein in *Litopenaeus vannamei* and study on its Agglutination characterization. J. Proteome. Res..

[8] Yan F., Zhang Y., Jiang R., Zhong M., Hu Z., Du H., Lun J., Chen J., Li Y. (2011). Identification and agglutination properties of hemocyanin from the mud crab (*Scylla serrata*). Fish Shellfish Immunol..

[9] Zheng L., Zhao X., Zhang P., Chen C., Liu S., Huang R., Zhong M., Wei C., Zhang Y. (2016). Hemocyanin from shrimp *Litopenaeus vannamei* has antiproliferative effect against HeLa cell in vitro. PLoS ONE.

[10] Zhang Y., Wang S., Peng X. (2004). Identification of a type of human IgG-like protein in shrimp *Penaeus vannamei* by mass spectrometry. J. Exp. Mar. Biol. Ecol..

[11] Zhang Y., Yan F., Hu Z., Zhao X., Min S., Du Z., Zhao S., Ye X., Li Y. (2009). Hemocyanin from shrimp *Litopenaeus vannamei* shows hemolytic activity. Fish Shellfish Immunol..

[12] Qiao J., Du Z., Zhang Y., Du H., Guo L., Zhong M., Cao J., Wang X. (2011). Proteomic identification of the related immune-enhancing proteins in shrimp *Litopenaeus vannamei* stimulated with vitamin C. and Chinese herbs. Fish Shellfish Immunol..

[13] Petit V.W., Rolland J.L., Blond A., Cazevieille C., Djediat C., Peduzzi J., Goulard C., Bachère E., Dupont J., Destoumieuxgarzón D. (2016). A hemocyanin-derived antimicrobial peptide from the penaeid shrimp adopts an alpha-helical structure that specifically permeabilizes fungal membranes. BBA-Gen Subjects.

[14] Destoumieuxgarzón D., Saulnier D., Garnier J., Jouffrey C., Bulet P., Bachère E. (2001). Crustacean immunity. Antifungal peptides are generated from the C terminus of shrimp hemocyanin in response to microbial challenge. J. Biol. Chem..

[15] Lee S.Y., Lee B.L., Söderhäll K. (2003). Processing of an antibacterial peptide from hemocyanin of the freshwater crayfish *Pacifastacus leniusculus*. J. Biol. Chem..

[16] Wen Y., Zhan S., Huang H., Zhong M., Chen J., You C., Wan F., Zhang Y.L. (2016). Identification and characterization of an 18.4 kDa antimicrobial truncation from shrimp *Litopenaeus vannamei* hemocyanin upon *Vibrio parahaemolyticus* infection. Fish Shellfish Immunol..

[17] Hancock R.E., Sahl H.G. (2006). Antimicrobial and host-defense peptides as new anti-infective therapeutic strategies. Nat. Biotechnol..

[18] Brogden K.A. (2005). Antimicrobial peptides: Pore formers or metabolic inhibitors in bacteria?. Nat. Rev. Microbiol..

[19] Bulet P., Stöcklin R., Menin L. (2004). Anti-microbial peptides: From invertebrates to vertebrates. Immunol. Rev..

[20] Zasloff M. (2002). Antimicrobial peptides of multicellular organisms. Nature.

[21] Gaspar D., Veiga A.S., Castanho M.A.R.B. (2013). From antimicrobial to anticancer peptides. A review. Front. Microbiol..

[22] Rodrigues E.G., Dobroff A.S., Cavarsan C.F., Paschoalin T., Nimrichter L., Mortara R.A., Santos E.L., Miranda A., Daffre S., Travassos L.R. (2008). Effective topical treatment of subcutaneous murine B16F10-Nex2 melanoma by the antimicrobial peptide gomesin. Neoplasia..

[23] Lin W.J., Chien Y.L., Pan C.Y., Lin T.L., Chen J.Y., Chiu S.J., Hui C.F. (2009). Epinecidin-1, an antimicrobial peptide from fish (*Epinephelus coioides*) which has an antitumor effect like lytic peptides in human fibrosarcoma cells. Peptides.

[24] Hilchie A.L., Doucette C.D., Pinto D.M., Patrzykat A., Douglas S., Hoskin D.W. (2011). Pleurocidin-family cationic antimicrobial peptides are cytolytic for breast carcinoma cells and prevent growth of tumor xenografts. Breast Cancer Res..

[25] Yang S., Huang H., Wang F., Aweya J.J., Zheng Z., Zhang Y. (2018). Prediction and characterization of a novel hemocyanin-derived antimicrobial peptide from shrimp *Litopenaeus vannamei*. Amino Acids.

[26] Coin I., Beyermann M., Bienert M. (2007). Solid-phase peptide synthesis: From standard procedures to the synthesis of difficult sequences. Nat. Protoc..

[27] Stark M., Liu L.P., Deber C.M. (2002). Cationic hydrophobic peptides with antimicrobial activity. Antimicrob. Agents Chemother..

[28] Koczulla A.R., Bals R. (2003). Antimicrobial peptides. Drugs.

[29] Li C., Zhu J., Wang Y., Chen Y., Song L., Zheng W., Li J., Yu R. (2017). Antibacterial activity of AI-Hemocidin 2, a novel *N*-terminal peptide of hemoglobin purified from *Arca inflata*. Mar. Drugs.

[30] Wang Y.H., Yu H.T., Pu X.P., Du G.H. (2013). Baicalein prevents 6-hydroxydopamine-induced mitochondrial dysfunction in SH-SY5Y cells via inhibition of mitochondrial oxidation and up-regulation of DJ-1 protein expression. Molecules.

[31] Adams J.M., Cory S. (1998). The Bcl-2 protein family: Arbiters of cell survival. Science.

[32] Shimizu S. (1999). Bcl-2 family proteins regulate the release of apoptogenic cytochrome c by the mitochondrial channel VDAC. Nature.

[33] Geller L.T., Barzily-Rokni M., Danino T., Jonas O.H., Shental N., Nejman D., Gavert N., Zwang Y., Cooper Z.A., Shee K. (2017). Potential role of intratumor bacteria in mediating tumor resistance to the chemotherapeutic drug gemcitabine. Science.

[34] García-Fernández E., Koch G., Wagner R.M., Fekete A., Stengel S.T., Schneider J., Mielich-Süss B., Geibel S., Markert S.M., Stigloher C. (2017). Membrane microdomain disassembly inhibits MRSA antibiotic resistance. Cell.

[35] Semina S.E., Scherbakov A.M., Vnukova A.A., Bagrov D.V., Evtushenko E.G., Safronova V.M., Golovina D.A., Lyubchenko L.N., Gudkova M.V., Krasil’nikov M.A. (2018). Exosome-mediated transfer of cancer cell resistance to antiestrogen drugs. Molecules.

[36] Rubinchik E., Dugourd D., Algara T., Pasetka C., Friedland H.D. (2009). Antimicrobial and antifungal activities of a novel cationic antimicrobial peptide, omiganan, in experimental skin colonisation models. Int. J. Antimicrob. Agents.

[37] Yeung A.T.Y., Gellatly S.L., Hancock R.E.W. (2011). Multifunctional cationic host defence peptides and their clinical applications. Cell Mol. Life Sci..

[38] Wang J., Wong E.S.W., Whitley J.C., Li J., Stringer J.M., Short K.R., Renfree M.B., Belov K., Cocks B.G. (2011). Ancient antimicrobial peptides kill antibiotic-resistant pathogens: Australian mammals provide new options. PLoS ONE.

[39] Standley S.M., Toft D.J., Cheng H., Soukasene S., Chen J., Raja S.M., Band V., Band H., Cryns V.L., Stupp S.I. (2010). Induction of cancer cell death by self-assembling nanostructures incorporating a cytotoxic peptide. Cancer Res..

[40] Hu J., Chen C., Zhang S., Zhao X., Xu H., Zhao X., Lu J.R. (2011). Designed antimicrobial and antitumor peptides with high selectivity. Biomacromolecules.

[41] Pushpanathan M., Gunasekaran P., Rajendhran J. (2013). Antimicrobial peptides: Versatile biological properties. Int. J. Pept..

[42] Guilhelmelli F., Vilela N., Albuquerque P., Derengowski L.D.S., Silvapereira I., Kyaw C.M. (2013). Antibiotic development challenges: The various mechanisms of action of antimicrobial peptides and of bacterial resistance. Front. Microbiol..

[43] Mulder K.C.L., Lima L.A., Miranda V.J., Dias S.C., Franco O.L. (2013). Current scenario of peptide-based drugs: The key roles of cationic antitumor and antiviral peptides. Front. Microbiol..

[44] Hoskin D.W., Ramamoorthy A. (2008). Studies on anticancer activities of antimicrobial peptides. BBA-Biomembranes.

[45] Guzmánrodríguez J.J., Ochoazarzosa A., Lópezgómez R., Lópezmeza J.E. (2015). Plant antimicrobial peptides as potential anticancer agents. Biomed. Res. Int..

[46] Li C., Wang F., Aweya J.J., Yao D., Zheng Z., Huang H., Li S., Zhang Y. (2018). Trypsin of *Litopenaeus vannamei* is required for the generation of hemocyanin-derived peptides. Dev. Comp. Immunol..

[47] Mader J.S., Hoskin D.W. (2006). Cationic antimicrobial peptides as novel cytotoxic agents for cancer treatment. Expert Opin. Investig. Drugs.

[48] Wang H., Ma J.L., Yang Y.G., Song Y., Wu J., Qin Y.Y., Zhao X.L., Wang J., Zou L.L., Wu J.F. (2016). Efficient therapeutic delivery by a novel cell-permeant peptide derived from KDM4A protein for antitumor and antifibrosis. Oncotarget.

[49] Green D.R., Galluzzi L., Kroemer G. (2011). Mitochondria and the autophagy-inflammation-cell death axis in organismal aging. Science.

[50] Earnshaw W.C., Martins L.M., Kaufmann S.H. (1999). Mammalian caspases: Structure, activation, substrates, and functions during apoptosis. Annu. Rev. Biochem..

[51] Thornberry N.A., Lazebnik Y. (1998). Caspases: Enemies within. Science.

[52] Nagata S. (2018). Apoptosis and clearance of apoptotic cells. Annu. Rev. Immunol..

[53] Man S.M., Kanneganti T.D. (2015). Converging roles of caspases in inflammasome activation, cell death and innate immunity. Nat. Rev. Immunol..

[54] Zhu L., Yuan H., Guo C., Lu Y., Deng S., Yang Y., Wei Q., Wen L., He Z. (2012). Zearalenone induces apoptosis and necrosis in porcine granulosa cells via a caspase-3- and caspase-9-dependent mitochondrial signaling pathway. J. Cell Physiol..

[55] Moldoveanu T., Follis A.V., Kriwacki R.W., Green D.R. (2014). Many players in BCL-2 family affairs. Trends Biochem. Sci..

[56] Green D.R., Reed J.C. (1998). Mitochondria and apoptosis. Science.

[57] Wang X., Chen C., Zhou G., Ye J., Yin R., Feng D., Zhang S., Wang X., Zhao X., Zhang Z. (2018). *Sepia* ink oligopeptide induces apoptosis of lung cancer cells via mitochondrial pathway. Cell Physiol. Biochem..

[58] Ow Y.P., Green D.R., Hao Z., Mak T.W. (2008). Cytochrome c: Functions beyond respiration. Nat. Rev. Mol. Cell Biol..

[59] Chipuk J.E., Fisher J.C., Dillon C.P., Kriwacki R.W., Kuwana T., Green D.R. (2008). Mechanism of apoptosis induction by inhibition of the anti-apoptotic BCL-2 proteins. Proc. Natl. Acad. Sci. USA.

[60] Kang M.H., Reynolds C.P. (2009). Bcl-2 Inhibitors: Targeting mitochondrial apoptotic pathways in cancer therapy. Clin. Cancer Res..

[61] Huertas N.J., Monroy Z.J.R., Medina R.F., Castañeda J.E.G. (2017). Antimicrobial activity of truncated and polyvalent peptides derived from the FKCRRQWQWRMKKGLA sequence against *Escherichia coli* ATCC 25922 and *Staphylococcus aureus* ATCC 25923. Molecules.

[62] Kong L.R., Chua K.N., Sim W.J., Ng H.C., Bi C., Ho J., Nga M.E., Pang Y.H., Ong W.R., Soo R.A. (2015). MEK inhibition overcomes cisplatin resistance conferred by SOS/MAPK pathway activation in squamous cell carcinoma. Mol. Cancer Ther..

[63] Shao Z.J., Zhang Y.Y., Fan Y.Y., Jin S.J., Yan J., Zheng X.W., Chen J., Yao X., Zhou L.M. (2012). β, β-Dimethylacrylshikonin exerts antitumor activity via Notch-1 signaling pathway in vitro and in vivo. Biochem. Pharmacol..

[64] Golks A., Brenner D., Schmitz I., Watzl C., Krueger A., Krammer P.H., Lavrik I.N. (2006). The role of CAP3 in CD95 signaling: New insights into the mechanism of procaspase-8 activation. Cell Death Differ..

